# Addressing the California Master Plan on Aging Through Innovative Gerontology Specializations

**DOI:** 10.1093/geroni/igab046.562

**Published:** 2021-12-17

**Authors:** Pamela Abbott-Enz

**Affiliations:** Chaffey College, Rancho Cucamonga, California, United States

## Abstract

The 2021 Master Plan for Aging outlines five bold goals to pursue over the next ten years, including addressing the issues of housing, health, and quality of life, finance, and caregiving, the plan also addresses public safety and emergency services, community programs and public spaces, access, inclusion, and equity. In order to prepare the workforce to meet these goals, California Community Colleges have the unique opportunity to collaborate and develop Aging Specialists in fields not traditionally age-focused. This presentation explores the possible scope and path toward curriculum development of five innovative collaborations that will prepare Gerontology specialists for the workforce that will be created through the execution of the California Master Plan.

